# Through-and-through access for mechanical aspiration thrombectomy of a catheter-associated internal jugular vein thrombosis mimicking lemierre syndrome

**DOI:** 10.1186/s42155-026-00772-x

**Published:** 2026-09-07

**Authors:** Francois H. Cornelis, Bruno Solis, Stephen B. Solomon

**Affiliations:** 1 Department of Radiology, Memorial Sloan Kettering Cancer Center, New York, NY 10065 USA; 2 Department of Medicine, Weill Cornell Medical College, New York, NY 10065 USA; 3 Department of Interventional Radiology, University of Miami, Miller School of Medicine, 1150 Northwest 14 Street, Miami, FL 33136 USA

To the Editor,

Internal jugular vein (IJV) thrombophlebitis refractory to anticoagulation and antibiotics remains a clinical challenge with limited evidence to guide interventional management [1]. A 57-year-old woman with newly diagnosed triple-negative breast cancer receiving neoadjuvant pembrolizumab, carboplatin, and paclitaxel presented with acute throat pain, neck swelling, dizziness, and a diffuse rash. Laboratory evaluation revealed leukopenia and anemia. Computed tomography of the neck demonstrated thrombophlebitis along the entire course of the right IJV, raising concern for Lemierre syndrome (Fig. 1A). A right-sided jugular vein single-lumen port had been placed 47 days prior. Multidisciplinary evaluation including head and neck surgery, rheumatology, and dermatology was obtained. The rash was attributed to immune checkpoint inhibitor toxicity, and rheumatology favored catheter-induced thrombophlebitis over vasculitis given the absence of systemic vasculitis features. The patient was initiated on intravenous piperacillin-tazobactam, vancomycin, metronidazole, and a therapeutic heparin infusion. Despite 72 h of appropriate antimicrobial therapy and systemic anticoagulation, the patient demonstrated persistent fever, worsening neck pain, and progressive swelling. Given lack of clinical improvement, the decision was made to proceed with mechanical aspiration thrombectomy (MAT) and concurrent port removal.

**Fig. 1:**
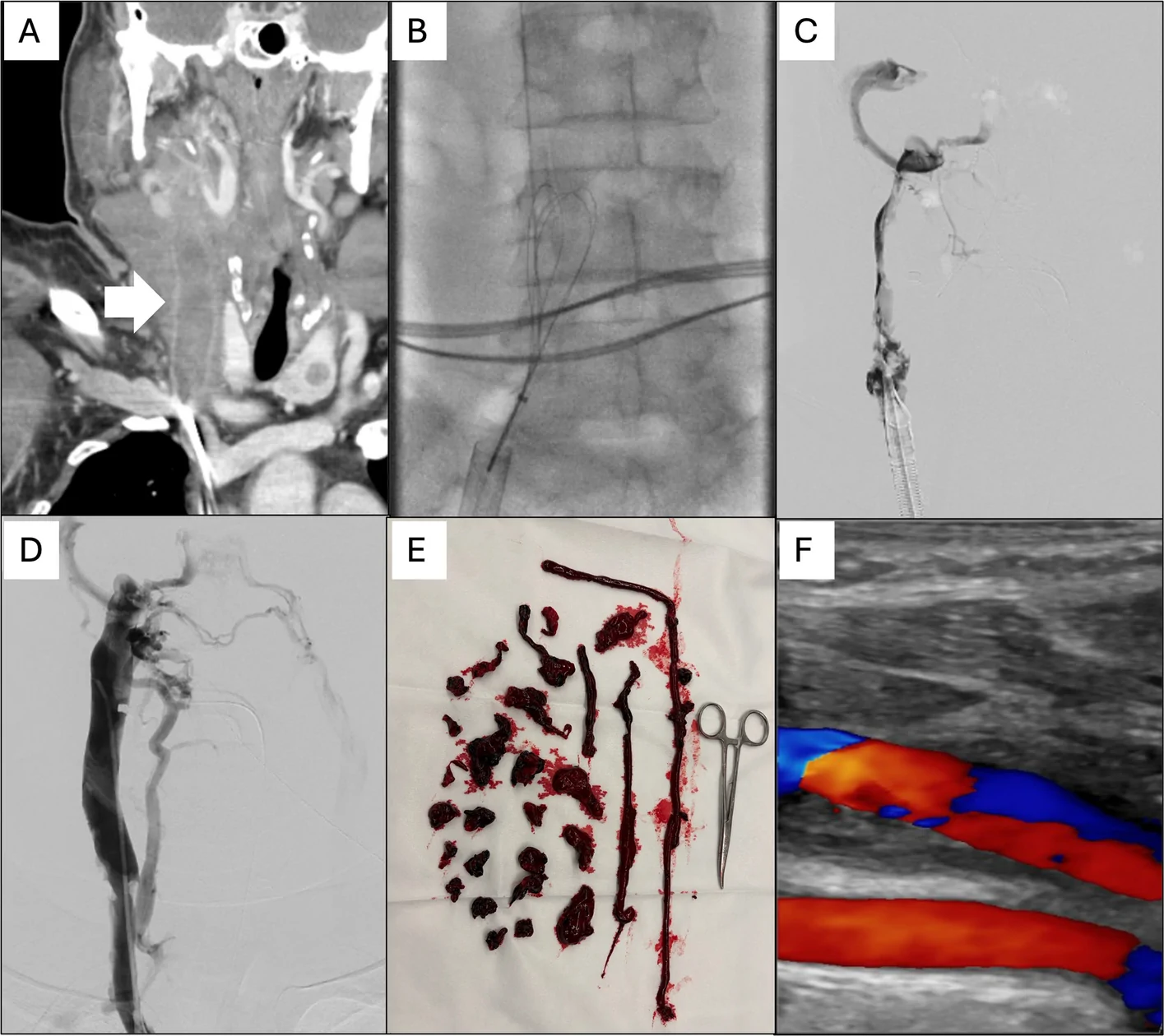
Mechanical aspiration thrombectomy of catheter-associated right internal jugular vein (IJV) thrombosis mimicking Lemierre syndrome in a 57-year-old woman with newly diagnosed triple-negative breast cancer receiving neoadjuvant pembrolizumab, carboplatin, and paclitaxel, presenting with acute throat pain, neck swelling, and persistent fever despite 72 h of antibiotics and anticoagulation. **A** Post contrast computed tomography of the neck (coronal view) demonstrating thrombophlebitis along the entire course of the right IJV (arrow) with an indwelling single-lumen port placed 47 days prior. **B** Through-and-through jugular-to-femoral rail, with a 0.035" guidewire advanced from the right IJV into the right femoral vein, where it was captured using a snare catheter to provide a stable platform across the completely occluded right IJV. **C** After diagnostic angiography confirmed complete occlusion of the right IJV, a mechanical aspiration thrombectomy was performed using the 24-F aspiration system positioned across the occluded segment. **D** Post-intervention angiography demonstrating restored patency of the right IJV from the jugular sinus to the superior vena cava, with minimal residual thrombus at the level of the previously removed port catheter tip. **E** Clots removed from the aspiration system. **F** Color flow Doppler ultrasound confirming restored venous flow in the right IJV following thrombectomy

This single case report was exempt from Institutional Review Board approval at the institution. Under general anesthesia, the port was removed prior to MAT. The right common femoral vein was accessed under ultrasound guidance using a micropuncture technique, and an 8-French sheath was placed, subsequently exchanged for a 24-French sheath. Given complete thrombotic occlusion of the right IJV preventing retrograde catheter navigation, a second 5-French access was obtained at the right IJV. A through-and-through rail was established: a 0.035-inch guidewire (Glidewire, Terumo, Somerset, NJ, USA) was advanced from the right IJV into the femoral vein, where it was captured using a snare catheter (Ensnare 27–45 mm, Merit Medical, South Jordan, UT, USA) through the femoral access (Fig. 1B). This provided a stable platform across the occluded segment, allowing safe accommodation of the MAT system. Diagnostic angiography via a 5-French Berenstein catheter confirmed complete IJV occlusion with permeability of the right lateral sinus. MAT was performed using a 24-French directional aspiration system (Aventus, Inquis Medical, Menlo Park, CA, USA) advanced over a 0.035-inch stiff guidewire (Advantage, Terumo, Somerset, NJ, USA) (Fig. 1C). Post-intervention angiography demonstrated restored patency from the jugular sinus to the superior vena cava, with minimal residual thrombus at the previously removed port catheter tip (Fig. 1D), confirmed by large clots collected from the device (Fig. 1E). Color flow Doppler ultrasound demonstrated restored venous flow (Fig. 1F). Intraprocedural heparin (3000 IU) was administered. Fluoroscopy time was 27 min and 56 s, reference air kerma was 288 mGy, kerma area product was 46.25 Gy·cm^2^, contrast volume was 90 mL of iodine contrast, estimated blood loss was less than 10 mL, total procedure time was 2 h and 22 min, and there were no immediate complications. Twelve hours post-thrombectomy, the patient developed transient dyspnea with a new oxygen requirement but CT angiography was negative for pulmonary embolism, and symptoms resolved spontaneously. By post-procedure day 5, the patient demonstrated marked clinical improvement with resolution of fever, dyspnea, and dizziness, and was breathing comfortably on room air. She was discharged on apixaban 5 mg twice daily for therapeutic anticoagulation, with a PICC line placed for completion of a 3-week course of intravenous daptomycin and piperacillin-tazobactam, given port tip cultures growing Corynebacterium species, although culture of clots remained negative.

Lemierre syndrome, characterized by IJV thrombophlebitis following oropharyngeal infection with an estimated incidence of 0.8 to 3.6 per million persons, has historically been managed with antibiotics and anticoagulation alone [2]. However, recent evidence suggests MAT may play a role in refractory cases, with rapid symptomatic improvement [3]. This case extends these observations to oncology patients with indwelling central venous catheters. When the IJV is completely occluded at the puncture site, standard retrograde wire navigation may be impossible, rendering conventional catheter-based thrombectomy unfeasible. Establishing a jugular-to-femoral through-and-through rail represents a technical solution that creates stable access for safe delivery of a large-bore aspiration catheter across an otherwise impassable segment. The risk of distal embolization during MAT in septic thrombus is low but justifies close cardiopulmonary monitoring post-thrombectomy. The transient post-procedural dyspnea prompted expedited imaging, ultimately negative for pulmonary embolism, reinforcing the need for vigilance without contraindicating the procedure [4].

In conclusion, the through-and-through jugular-to-femoral rail technique described here provides a reproducible strategy for interventional radiologists facing complete IJV occlusion, offering a safe and effective means to deliver large-bore aspiration systems when conventional antegrade or retrograde access fails. Combined with concurrent source removal, MAT may reduce morbidity and hospitalization compared to prolonged conservative management, supporting its consideration when patients fail to improve after 48 to 72 h of appropriate therapy.

## Data Availability

All data generated or analyzed during this study are included in this published article.

## Abbreviations

CT: Computed tomography
IJV: Internal jugular vein
MAT: Mechanical aspiration thrombectomy
PICC: Peripherally inserted central catheter

## References

[1] Scerrati A, Menegatti E, Zamboni M, Malagoni AM, Tessari M, Galeotti R, et al. Internal jugular vein thrombosis: etiology, symptomatology, diagnosis and current treatment. Diagnostics (Basel). 2021;11(2):378. 10.3390/diagnostics11020378. 33672254 PMC7926529

[2] Min SK, Park YH, Cho YK, Park JW, Koh YH, Seo TS. Lemierre’s syndrome: unusual cause of internal jugular vein thrombosis--a case report. Angiology. 2005;56(4):483–7. 10.1177/000331970505600417. 16079933

[3] Davis J, Adams C, Gupta P, Samant S, Marcinkowski B, Edwards S, et al. Thrombectomy in Lemierre’s syndrome: a systematic literature review. Iran J Otorhinolaryngol. 2026;38(2):75–81. 10.22038/ijorl.2025.91192.4043. 42006902 PMC13090815

[4] Valerio L, Zane F, Sacco C, Granziera S, Nicoletti T, Russo M, et al. Patients with Lemierre syndrome have a high risk of new thromboembolic complications, clinical sequelae and death: an analysis of 712 cases. J Intern Med. 2021;289(3):325–39. 10.1111/joim.13114. 32445216

